# Vasopressors and Risk of Acute Mesenteric Ischemia: A Worldwide Pharmacovigilance Analysis and Comprehensive Literature Review

**DOI:** 10.3389/fmed.2022.826446

**Published:** 2022-05-23

**Authors:** Mathieu Jozwiak, Guillaume Geri, Driss Laghlam, Kevin Boussion, Charles Dolladille, Lee S. Nguyen

**Affiliations:** ^1^Service de Médecine Intensive Réanimation, Centre Hospitalier Universitaire l'Archet 1, Nice, France; ^2^Equipe 2 CARRES UR2CA—Unité de Recherche Clinique Côte d'Azur, Université Côte d'Azur UCA, Nice, France; ^3^Service de Médecine Intensive Réanimation, Centre Médico-Chirurgical Ambroise Paré, Neuilly-sur-Seine, France; ^4^Faculté de Médecine, Université de Paris, Paris, France; ^5^Department of Pharmacology, CHU de Caen, Caen, France

**Keywords:** septic shock (MeSH), vasopressors, pharmacovigilance, systematic review, mesenteric ischemia

## Abstract

Vasodilatory shock, such as septic shock, requires personalized management which include adequate fluid therapy and vasopressor treatments. While these potent drugs are numerous, they all aim to counterbalance the vasodilatory effects of a systemic inflammatory response syndrome. Their specific receptors include α- and β-adrenergic receptors, arginine-vasopressin receptors, angiotensin II receptors and dopamine receptors. Consequently, these may be associated with severe adverse effects, including acute mesenteric ischemia (AMI). As the risk of AMI depends on drug class, we aimed to review the evidence of plausible associations by performing a worldwide pharmacovigilance analysis based on the World Health Organization database, VigiBase®. Among 24 million reports, 104 AMI events were reported, and disproportionality analyses yielded significant association with all vasopressors, to the exception of selepressin. Furthermore, in a comprehensive literature review, we detailed mechanistic phenomena which may enhance vasopressor selection, in the course of treating vasodilatory shock.

## Background

Vasodilatory shock, among which the first cause is septic shock, rank first in the causes of mortality in intensive care units (1). Upkeeping organs perfusion is one of the main goals of shock management, and to that end, volume resuscitation and vasopressors administration are key to maintaining adequate blood pressure (1).

Yet, the variety of molecules which may be used as vasopressors has been increasing over the years, and their efficacy and drawbacks have been compared in numerous meta-analyses and reviews. Among adverse effects related to use of vasopressors, acute mesenteric ischemia (AMI) is an uncommon (with a below than 1% incidence) but lethal (2, 3) and its diagnosis difficult (4).

In this review, we especially focused on AMI related to vasopressors use. In the first part, we performed a worldwide pharmacovigilance analysis based on the World Health Organization database, VigiBase® to assess the potential association between the different vasopressors and AMI. In the second part, we performed a comprehensive literature review on the mechanisms of action of the available vasopressors at bedside and their respective adverse effects, with a focus on AMI.

## Methods

### Study Design

This is a worldwide observational case-non-case cross-sectional study focusing on AMI related to vasopressors use, from the international pharmacovigilance database, VigiBase^®^ (5). VigiBase^®^ is the WHO global individual case safety reports (ICSR) deduplicated database, managed by the Uppsala-Monitoring-Centre (Uppsala, Sweden, accessible at www.vigiaccess.org). It contains over 23 million ICSR received from over 130 countries since 1967 with over 25,000 drugs and vaccines. ICSR originate from different sources, such as healthcare professionals, patients, and pharmaceutical companies, and are generally notified post-marketing. ICSR include administrative information (country, type of report and reporter), patient data (age, sex) and nature of the outcome, using the latest version (currently v22.1) of MedDRA (Medical Dictionary for Regulatory Activities) terms (6). Drug(s) involved (name, drug start and stop dates, indication, dose) are also indicated. Drugs are coded using the WHO drug dictionary and categorized using the Anatomical Therapeutic Chemical (ATC) classification (7). Each event is characterized as “serious” or “non-serious” according to the WHO definition. Seriousness corresponds to death, life-threatening situations, hospitalization, hospitalization prolongation, persistent incapacity or disability, and situations judged clinically serious by the physician reporting the case.

### Analysis in VigiBase^®^

VigiBase^®^ is a spontaneous reporting system, which allows for more robust and rigorous analyses than isolated case reports or case series, due to the possibility of performing quantitative comparisons, such as disproportionality analysis (case–non-case) to identify drugs significantly associated with AMI (8). We identified cases of AMI by searching in VigiBase^®^ all ICSR flagged with the MedDRA preferred-term level referring to AMI (a composite of “*Intestinal ischemia*,” “*Mesenteric arterial occlusion*,” “*Mesenteric artery stenosis*,” “*Mesenteric vascular insufficiency*” and “*Mesenteric vascular occlusion*”) from inception to June 2021; with a drug declared as “suspect” or “interacting” with AMI reaction.

Disproportionality analysis compares the proportion of a selected specific adverse-drug-reaction (ADR) reported for a single drug with the proportion of the same ADR for a control group of drugs (i.e., full database with all drugs). The denominator in these analyses is the total number of ADR reported for each group of drugs. If the proportion of cases associated with a specific drug is greater than in patients without this ADR (non-cases), there is a disproportionality association (signal identification) between the ADR and the drug. In the present work, the calculated Bayesian disproportionality estimate was the information component (IC) (8). Herein, we also performed for selected previously unknown liable drugs a sensitivity analysis excluding from full database the ICSR in which drugs already known to be associated with AMI were reported.

Calculation of the IC using a Bayesian confidence propagation neural network was developed and validated by the Uppsala Monitoring Centre as a flexible, automated indicator value for disproportionate reporting that compares observed and expected ADR associations to find new drug-ADR signals with identification of probability difference from the background data (full database) (9). Probabilistic reasoning in intelligent systems (information theory) has proved to be effective for the management of large datasets, is robust in handling incomplete data, and can be used with complex variables. The information theory tool is ideal for finding drug-ADR combinations with other variables that are highly associated compared with the generality of the stored data (9). Several examples of validation with the IC exist, showing the power of the technique to find signals sooner after drug approval than by a regulatory agency, and to avoid false positives, whereby an association between a common drug and a common ADR occurs in the database only because the drug is widely used and the ADR is frequently reported (i.e., between digoxin and rash) (9, 10). Like others, our team published several studies using VigiBase^®^ and disproportional reporting calculation to characterize and identify new drug-ADR associated signals, which were subsequently corroborated by preclinical mechanistic studies or prospective cohorts (8, 11–14). This later element requires to be emphasized, as IC value should be interpreted only as means to perform clinical reviews of plausible associations and do not signify causality in any way. The IC_025_ is the lower end of the 95% credibility interval for the IC. A positive value of the IC_025_ is deemed significant (8, 15).

For description of ICSR, continuous data were reported in median (interquartile range). All data were available, otherwise specified. Data management was performed using Python software v3.0 (Python software foundation, Wilmington, Delaware, USA).

## Results

**Figure 1:**
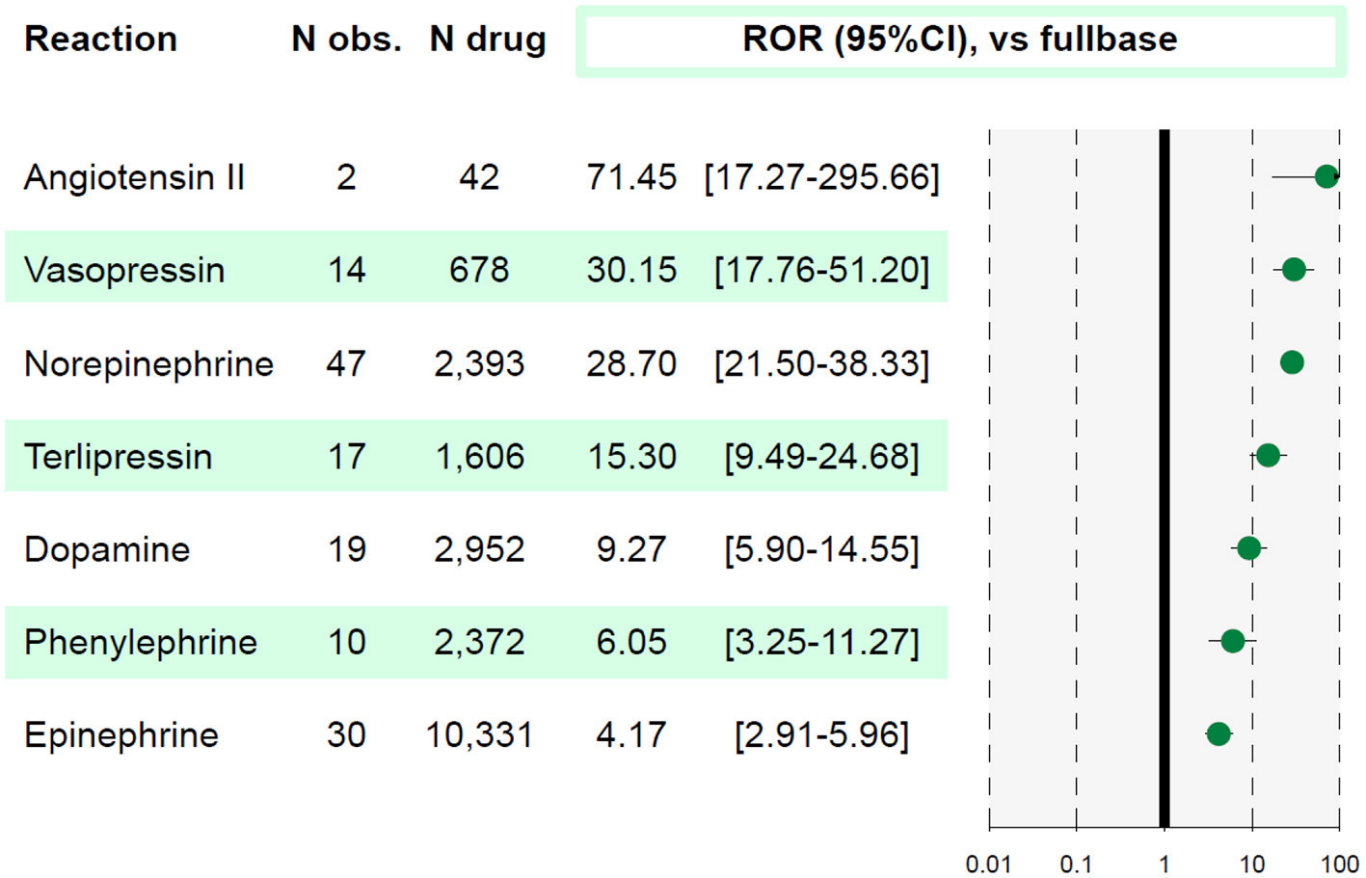
Association between acute mesenteric ischemia and each molecule. A reporting odds ratio (ROR) value is considered significant when lower bound of 95% confidence interval (95%CI) is above 1. The studied reaction is acute mesenteric ischemia (AMI). *N obs*., number of observed AMI reports; *N drug*, number of reports involving the studied drug in VigiBase^®^.

Overall, 23,937,083 ICSR were screened and 104 ICSR of AMI were retained. All vasopressors, to the exception of selepressin were significantly associated with AMI (IC_025_ > 0 and ROR > 1, see Figure 1). Affected patients were >65 years old in 48%, with men representing 61% of cases. Death was concomitantly reported in 49% of cases. Reports originated from standard of care in 91% and 6 from investigational drug studies. The summary of ICSR characteristics by vasopressor is detailed in Table 1.

**Table 1:** Descriptive statistics by molecule of all reports of acute mesenteric ischemia in VigiBase^®^.

**Drug of interest**	**Overall**	**Norepinephrine**	**Epinephrine**	**Phenylephrine**	**Dopamine**	**Vasopressin**	**Terlipressin**	**Angiotensin II**
Nb of cases	104	47	30	10	19	14	17	2
Male	59 (60.8%) [97]	28 (59.6%) [47]	15 (53.6%) [28]	3 (30.0%) [10]	10 (62.5%) [16]	9 (69.2%) [13]	9 (64.3%) [14]	2 (100.0%) [2]
Age > 65 years-old	44 (47.8%) [92]	24 (53.3%) [45]	15 (53.6%) [28]	3 (37.5%) [8]	7 (43.8%) [16]	3 (25.0%) [12]	5 (35.7%) [14]	1 (50.0%) [2]
Serious adverse event	96 (100.0%) [96]	46 (100.0%) [46]	30 (100.0%)	7 (100.0%) [7]	17 (100.0%) [17]	14 (100.0%)	14 (100.0%) [14]	2 (100.0%)
Deaths	47 (49.0%) [96]	22 (47.8%) [46]	15 (50.0%)	6 (85.7%) [7]	8 (47.1%) [17]	9 (64.3%)	8 (57.1%) [14]	0 (0.0%)
**Region of reporting**								
Africa	1 (1.0%)	0 (0.0%)	0 (0.0%)	0 (0.0%)	1 (5.3%)	0 (0.0%)	0 (0.0%)	0 (0.0%)
America	27 (26.0%)	10 (21.3%)	12 (40.0%)	3 (30.0%)	8 (42.1%)	7 (50.0%)	0 (0.0%)	2 (100.0%)
South-East Asia	0 (0.0%)	0 (0.0%)	0 (0.0%)	0 (0.0%)	0 (0.0%)	0 (0.0%)	0 (0.0%)	0 (0.0%)
Europe	42 (40.4%)	23 (48.9%)	12 (40.0%)	5 (50.0%)	1 (5.3%)	2 (14.3%)	12 (70.6%)	0 (0.0%)
East Meditterranean	0 (0.0%)	0 (0.0%)	0 (0.0%)	0 (0.0%)	0 (0.0%)	0 (0.0%)	0 (0.0%)	0 (0.0%)
West Pacific	34 (32.7%)	14 (29.8%)	6 (20.0%)	2 (20.0%)	9 (47.4%)	5 (35.7%)	5 (29.4%)	0 (0.0%)
**Type or reporting**								
Spontaneous	94 (91.3%) [103]	42 (89.4%) [47]	27 (90.0%) [30]	9 (90.0%) [10]	18 (94.7%) [19]	13 (92.9%) [14]	14 (87.5%) [16]	2 (100.0%) [2]
Report from study	6 (5.8%) [103]	4 (8.5%) [47]	2 (6.7%) [30]	0 (0.0%) [10]	1 (5.3%) [19]	0 (0.0%) [14]	2 (12.5%) [16]	0 (0.0%) [2]
Other	3 (2.9%) [103]	1 (2.1%) [47]	1 (3.3%) [30]	1 (10.0%) [10]	0 (0.0%) [19]	1 (7.1%) [14]	0 (0.0%) [16]	0 (0.0%) [2]
**Category of reporter**								
Physician	75 (79.8%) [94]	34 (82.9%) [41]	21 (75.0%) [28]	4 (66.7%) [6]	14 (93.3%) [15]	6 (54.5%) [11]	15 (88.2%) [17]	1 (50.0%) [2]
Pharmacist	2 (2.1%) [94]	1 (2.4%) [41]	0 (0.0%) [28]	0 (0.0%) [6]	0 (0.0%) [15]	1 (9.1%) [11]	0 (0.0%) [17]	0 (0.0%) [2]
Other health professional	13 (13.8%) [94]	6 (14.6%) [41]	6 (21.4%) [28]	2 (33.3%) [6]	0 (0.0%) [15]	4 (36.4%) [11]	0 (0.0%) [17]	0 (0.0%) [2]
Lawyer	0 (0.0%) [94]	0 (0.0%) [41]	0 (0.0%) [28]	0 (0.0%) [6]	0 (0.0%) [15]	0 (0.0%) [11]	0 (0.0%) [17]	0 (0.0%) [2]
Consumer or non-health professional	4 (4.3%) [94]	0 (0.0%) [41]	1 (3.6%) [28]	0 (0.0%) [6]	1 (6.7%) [15]	0 (0.0%) [11]	2 (11.8%) [17]	1 (50.0%) [2]

*Number of available data are indicated in brackets*.

## Discussion and Review of the Different Available Vasopressors

Vasopressors are indicated for patients with persistent arterial hypotension after appropriate fluid resuscitation. While some vasopressors are natural hormones that exert a vasopressor activity through specific receptor activation (norepinephrine, epinephrine, vasopressin, angiotensin II), most recent vasopressors, such as selepressin, are modifications of natural hormones. All vasopressors have adverse effects such as ischemia, cardiac arrhythmias and/or metabolic changes. Here, we confirmed in this worldwide pharmacovigilance study, an association between use of all vasopressors and AMI, to the exception of selepressin.

Pharmacovigilance disproportionality analyses using IC and ROR have long been considered relevant toward building case for delving deeper into associations between incriminated drugs and specific ADR, using spontaneous reports as material. As for any other measures of disproportionality, the need for caution to interpret quantitative results is paramount and IC values primarily serve to triage which drugs or drug categories require scrutiny while building case reviews (16). Hence, the primary aim of such methods is to look at plausible drug-ADR associations, before delving deeper using combined *in vitro* and *in vivo* translational methods to assess causality (13).

### Gastrointestinal Side Effects of Vasopressors, an Overview

In critically-ill patients with shock, vasopressors are used to restore vasoconstriction and enhancement of mean arterial pressure. However, a higher cumulative vasopressor dose is associated with organ dysfunction and mortality (17, 18). Vasopressors are also associated with digestive side effects when used inappropriately or in high doses.

Gastrointestinal complications are frequent in critically ill patients (19). Mechanisms underlying vasopressors use and gastrointestinal complications are not fully understood yet, but splanchnic blood flow seems to be a major factor. Firstly, the restoration of microcirculatory blood flow is not distributed evenly when vasopressors are used, especially in the digestive organs. In pigs who were exposed to fecal peritonitis-induced septic shock; norepinephrine and epinephrine failed to increase microcirculatory blood flow in most abdominal organs, despite increased perfusion pressure and systemic blood flow (20). These both drugs appeared to divert blood flow away from the mesenteric circulation and decrease microcirculatory blood flow in the jejunal mucosa and pancreas (20). In late 90s, the effects of vasopressors on increasing the splanchnic perfusion, principally assessed by gastric intramucosal pH, was found to be unpredictable (21). Secondly, in critically-ill patients, the use of catecholamines and degree of motility disturbance were found to be associated (22), although the severity of illness and use of sedative drugs disturbs motility and also associates with catecholamine use (22).

Splanchnic vasoconstriction, secondary to the vasopressors use, could lead to non-occlusive AMI, characterized by gastrointestinal ischemia with “normal” vessels. Overall, non-occlusive AMI is associated with a high mortality rate in critically-ill patients (2, 23). The mechanisms underlying non-occlusive AMI are incompletely understood and include macrovascular vasoconstriction, hypoperfusion of the tips of the villi and shunting (4). However, causality link between vasopressors use and AMI was not established on randomized studies (24).

### Angiotensin II

The main angiotensin II cardiovascular effects are the regulation of arterial blood pressure with short-term vasoconstriction, the regulation of aldosterone synthesis and vasopressin release and the regulation of the water and salt balance. All these effects are primary mediated through the binding of angiotensin II to its type 1 receptor, which belongs to the G protein-coupled receptor superfamily, in blood vessels, kidney, brain and heart (25). Besides its classical cardiovascular effects, angiotensin II might also exert inflammatory, pro-proliferative and pro-fibrotic effects, involved in oncologic and transplantation pathways (26).

Given its vasopressor activity which allows the restoration of vascular tone and arterial pressure through both venous and arterial constriction, (27) the interest of angiotensin II administration has been studied in vasodilatory shock and especially in septic shock, since a relative renin-angiotensin system failure has been evidenced in sepsis, illustrated by a relative decrease in angiotensin II plasma levels combined with a decrease in sensitivity to angiotensin II stimulation (26). Thus, some experimental (28–30) and human studies (31–35) have shown that angiotensin II administration allowed improvements to arterial pressure and even a catecholamine-sparing effect (36). In 2017, the multicentric randomized double-blind, placebo-controlled ATHOS-3 trial conducted in 334 patients with high-output catecholamine-resistant vasodilatory shock, defined by persistent vasodilatory shock despite adequate fluid resuscitation and administration of high doses of norepinephrine for a minimum of 6 h and a maximum of 48 h, showed that low-dose of angiotensin II allowed to achieve a predefined mean arterial pressure target along with a decrease in catecholamine dosage, but did not reduce the mortality rate (37). *Post-hoc* analyses showed that patients who benefited most from angiotensin II administration were those with the most severe shocks with a relative angiotensin II deficiency, (38) those with markedly elevated serum renin concentrations at baseline (39) and those with acute kidney injury requiring renal replacement therapy (40). In this latter group of patients, the administration of angiotensin II was associated with a lower 28-day mortality rate, a better correction of hypotension, and a faster recovery of kidney function (40).

Despite these encouraging results, angiotensin II is currently not recommended in patients with septic shock (1), since its safety is still matter of debate. Indeed, the marked vasopressor activity of angiotensin II could result in AMI and microvascular thrombosis in experimental models of septic shock (41, 42). Nonetheless, in the ATHOS-3 trial, the proportion of serious ischemic adverse effects (digital, gut, myocardial) and cardiac arrhythmias were similar in patients receiving angiotensin II or placebo (37). Especially, AMI occurred in <1% of patients receiving angiotensin II (37). A systematic review also concluded that angiotensin II-induced side effects were infrequent, with ≤ 300 reported adverse effects, and no AMI was reported (43). It must be noted that only 13 of the included studies were conducted in patients with vasodilatory shock, making the external validity of these results questionable in the case of critically-ill patients.

### Non-catecholaminergic Vasopressors: Vasopressin, Selepressin, Terlipressin

Non-catecholaminergic vasopressors rely on an alternate pathways, which all depend on three receptors, responsive to plasma osmolality, blood volume and pressure (44, 45):

- V1a receptors, located on vascular smooth muscular cells, allowing vasoconstriction,
- V1b receptors, mostly located in the anterior-pituitary gland and in the pancreas. Their activation leads to the endocrinological role of vasopressin (AVP), especially by the induction of corticotropic axis stimulation,
- V2 receptors, located on basolateral surface of renal tubular cells, inducing aquaporin 2 recruitment leading to water reabsorption.

AVP has pleiotropic effects, by stimulating all three receptors. Selepressin is a selective agonist of V1a receptors, which may mitigate sepsis-induced vasodilatation, vascular leakage and tissue edema. Finally, terlipressin is mostly a V1a receptor agonist, but also interacts with V1b and V2 receptors. To be noted, selepressin and terlipressin use are justified by the potential toxicity of V2 activity on endothelial and renal cells of vasopressin (46).

#### Vasopressin (AVP)

AVP is a nine-amino-acid peptide that is produced by the hypothalamus and then stored in the post-pituitary gland. Its vasoconstrictive effect is very low in healthy individuals (47, 48). In patients with vasodilatory shock such as septic shock, AVP has a much more potent effect and is released at the very early phase of shock (49). Its effect is then potentiated within the first 2 h after septic injury (i.e., endotoxinemia), with an increase in plasma levels concentration (50). Interestingly, if the injury lasts more than a few hours, the levels of AVP drop under baseline level, leading to a hormonal paradoxical level that can be observed during critical illnesses (51). This decrease may be due to the depletion of pituitary stores (52), although, only a third showed this feature in a cohort of patients with septic shock (53). Autonomic dysfunction with impaired baroreflex loop and osmoregulation may also participate to the low levels of AVP observed during septic shock (52–56). Furthermore, increased neuronal apoptosis in the autonomic centers may also contribute to the observed deficiency in AVP during septic shock (57, 58).

In contrast to norepinephrine, AVP may cause less vasoconstriction in mesenteric, coronary, and cerebral circulations (59). In several *in vivo* models, AVP was associated with improved recovery from mesenteric ischemia. The upregulation of endothelin 1 (ET-1) gene expression, with subsequent increased plasma levels of ET-1 and intestinal fatty acid binding protein, has been previously associated with mesenteric ischemia, and use of vasopressin in porcine models reversed these observations (60). Interestingly, endothelin receptor antagonists have been identified as potential protectors against ischemia-reperfusion injury in small intestine, and it was suggested in rats that AVP may have cross-path effect (61).

The largest trial compared in 778 patients with septic shock, the effect of vasopressin to those of norepinephrine (62). There was no difference in overall survival (35.4 and 39.3% 28-day mortality, respectively; difference, −3.9%; 95% CI, −10.7 to 2.9), however, the less severe patients who received AVP tended to show lower mortality, but interaction tests between severity of shock and mortality did not confirm this observation. AMI occurred in similar proportion in both treatment arms (3.4% in the norepinephrine arm vs. 2.3% the AVP arm, *p* = 0.39). In a recent meta-analysis, AVP use in septic shock was associated with an increase of digital ischemia (RR 4.85, 95% CI 2.81–8.39, I_2_ = 26%), but not AMI (RR 0.83, 95% CI 0.44–1.55, I_2_ = 0%) and diarrhea (2.47, 95% CI 0.77–7.96, I_2_ = 49%) (63). Hence, to date, no prospective studies in human showed differences in AMI when comparing AVP to another vasopressor, but the scarcity of data does not allow any conclusion, yet. Currently AVP is recommended as a second-line vasopressor in patients with septic shock (1).

#### Terlipressin

Terlipressin is mainly used in patients with hepatic failure, hepatorenal syndrome (64), and esophageal varices rupture bleeding (65). Only few trials assessed the benefits of terlipressin compared to norepinephrine in vasodilatory shock. Moreover, terlipressin was either evaluated alone or in combination with other vasopressors, with various dosage, and patients' profiles.

The largest randomized controlled trial comparing terlipressin and norepinephrine in patients with septic shock was stopped due to futility (66). In 526 patients, neither difference in 28-day mortality was observed nor vasopressor-free days or change in SOFA score during the first week after randomization. While the investigators reported a greater prevalence of adverse effects (30 vs. 12%, *p* < 0.01) including diarrhea in the terlipressin group, they did not find more AMI (1.02 vs. 0.35%, *p* = 0.62) (66).

Since then, several meta-analyses were published. In 2019, Huang et al. (67) assessed only randomized controlled trials specifically evaluating terlipressin vs. norepinephrine, each in single-therapy in the management of septic shock. With 6 studies included in their analysis, the authors showed no difference in 28-day mortality, urine output, liver and kidney functions as well as in adverse effects between groups (67). More recently, Yao et al. (68) compiled trials evaluating terlipressin alone or with norepinephrine compared with norepinephrine alone or with dopamine. They observed a significant lower 28-day or 30-day mortality rate among patients with septic shock who received terlipressin [RR = 0.87 (95%CI, 0.77–0.98)]. Adverse effects, including AMI, were not different between groups (68).

#### Selepressin

Selepressin is a pure V1a agonist. *In vitro* and *in-vivo* studies on animals showed it can reduce endothelial barrier dysfunction, vasodilatation, capillary leakage, lung edema and pro-inflammatory cytokines generated by sepsis (69–73).

Only two major studies focused on selepressin in addition to norepinephrine in patients with septic shock. A phase IIa trial highlighted that patients receiving selepressin compared to placebo, received less catecholamine while maintaining mean arterial pressure and reducing net fluid balance (74). A larger study, SEPSIS-ACT, published in 2019 by Laterre et al. (75), evaluated the value of adding to norepinephrine, selepressin compared to placebo in patients with septic shock in a phase 2b/3 trial. The trial was stopped for futility, finding no difference on the primary endpoint: ventilator- and vasopressor-free days within 30 days nor in any of the secondary end points (90-day mortality, kidney replacement therapy-free days, intensive care unit-free days). However, beneficial effects were observed in the selepressin group: decreased norepinephrine doses and positive fluid balance, increased urine output. Focusing on adverse effects, the proportion of AMI was no greater in the selepressin group than in the placebo group (3.2 vs. 2.6%) (75).

### Catecholaminergic Vasopressors: Norepinephrine, Epinephrine, Phenylephrine and Dopamine

#### Norepinephrine

Norepinephrine is a potent α- and β1-adrenergic agonist, with little activity on β2 receptors. By binding to its receptors, norepinephrine increases cytosolic calcium concentration into smooth muscle, leading to vasoconstriction and some positive inotropic activity. Through its β-adrenergic effect, norepinephrine exerts its vasopressor activity with arterial and venous vasoconstriction. Besides the increase in arterial pressures, left ventricular afterload and cardiac filling pressures, norepinephrine also increases the venous return, resulting in an increase in right atrial pressure and cardiac preload (76, 77). This increase in venous return results from the increase in mean systemic filling pressure (78, 79) and thus in venous return pressure gradient. Through its β1 stimulation, norepinephrine also exerts a positive inotropic effect and an increase in stroke volume. It has been recently demonstrated in 38 patients with septic shock who had been resuscitated for <3 h and whose mean arterial pressure remained <65 mmHg, in whom norepinephrine administration increased the left and right systolic function and the cardiac output despite the increased left ventricular afterload (76, 77, 80). Interestingly, the potential chronotropic effect is counteracted by baroreflex stimulation following vasoconstriction. Consequently, norepinephrine increases cardiac output without increasing heart rate or myocardial oxygen consumption (81). Finally, norepinephrine enhances the coronary blood flow because of coronary vasodilation secondary to enhanced cardiac metabolism and the normalization of diastolic blood pressure when low.

Besides to its effects on macrocirculation, norepinephrine administration might also improve microcirculation, especially in case of septic shock, which is characterized by microcirculatory abnormalities even in patients with preserved or corrected microcirculation (82). To this end, Georger et al. (83) demonstrated in severely hypotensive patients with septic shock that norepinephrine administration improved muscle tissue oxygenation and microcirculatory reserve capacities. In addition, the assessment of tissue oxygenation might be of interest to personalize mean arterial pressure target and thus the dosage of norepinephrine in patients with septic shock (84). Finally, it has also been suggested that norepinephrine might have some immune effects (85).

While norepinephrine is currently recommended as the first-line vasopressor in patients with septic shock (1), hemorrhagic shock (86), and cardiogenic shock (87–89), some potential adverse effects of high-dose of norepinephrine should be nonetheless kept in mind. First, high-dose of norepinephrine may induce oxidative stress and myocardial cells insult but also alter sepsis-associated immunomodulation (90). Furthermore, high-dose of norepinephrine may impair the splanchnic circulation with an increase in systemic and mesenteric vascular resistances (91). Nevertheless, a large randomized trial, the SEPSISPAM study, assessed the effects of two levels of mean arterial pressure in 776 patients with septic shock (92). To achieve the high mean arterial pressure level, norepinephrine doses were significantly increased. The 28-day mortality rate was not different between both groups. Serious adverse effects related to norepinephrine use were ventricular arrhythmias, bleeding, as well as digital ischemia and AMI. Except atrial fibrillation which was more frequent in patients receiving higher dose of norepinephrine, the incidence of other adverse effects, including AMI, was similar in both groups of patients and AMI occurred in 2% of patients (92).

#### Epinephrine

Epinephrine is the first adrenergic hormone of the adrenal medullar gland which was identified and is a potent agonist of α, β1 and β2 receptors. Through its α-adrenergic effect, epinephrine exerts its vasopressor activity with marked arterial and venous vasoconstriction. However, the epinephrine effects on vasculature is partly counteracted by β2-mediated vasodilation. Thus, epinephrine administration results clinically in a marked increase in systolic arterial pressure while diastolic arterial pressure only slightly increased. Consequently, the increase in mean arterial pressure is less than that with norepinephrine. Through its β1 stimulation, which is more marked than that of norepinephrine (81, 93), epinephrine also exerts positive inotropic and chronotropic effects, resulting in an increase in cardiac output. Epinephrine also facilitates ventricular relaxation and enhanced coronary blood flow through the increase in myocardial oxygen consumption. Finally, as with norepinephrine, it has also been suggested that epinephrine might have some immune effects (85).

Two large trials evaluated the effects of epinephrine administration in critically-ill patients (94, 95). In the CAT study, Myburgh et al. showed in 280 patients with shock (mainly septic shock) that the median time to achieve a predefined mean arterial pressure target was similar with epinephrine and norepinephrine administration. The 28- and 90-day mortality rate was also similar and there was no difference in vasopressor-free days (94). However, epinephrine administration was associated with more frequent lactic acidosis and arrhythmia, which led to the discontinuation of the administration of epinephrine in 13% of patients. Of note, no AMI or others ischemic adverse effects were reported (94). In the multicentric and randomized CATS study, Annane et al. (95) compared in 330 patients with septic shock epinephrine alone to the association norepinephrine and dobutamine. The different mortality rates were not different as the time to achieve hemodynamic success and the time to vasopressors withdrawal (95). Once again, epinephrine was associated with more frequent lactic acidosis, but the incidence of the other severe adverse effects (arrhythmias, ischemic events, bleeding) was similar between epinephrine and the association norepinephrine and dobutamine and AMI was reported (95). Epinephrine-induced lactic acidosis is a well-known metabolic effect (96, 97), which is assumed to be independent of tissue hypoxia and related to the activation of the β2-adrenergic receptors located at the surface of the skeletal muscle cells (98). This β2-activity stimulates the skeletal muscle cell Na+/K+-ATPase and accelerates the aerobic glycolysis and thus the production of pyruvate and hence of lactate into the cell (99).

Regarding the microcirculation, while it has been suggested in patients with septic shock that epinephrine administration increased more gastric mucosal perfusion than norepinephrine alone for the same mean arterial pressure level (100), other experimental (20, 101) and human studies (96, 100, 102–104) suggested that epinephrine might impair splanchnic circulation. Finally, compared to other vasopressors, epinephrine has the most negative inhibitory effect of propulsive gut motility (105). Thus, because of its more marked metabolic and cardiac adverse effects than norepinephrine (81, 93), and its potential deleterious effects on microcirculation, epinephrine is currently considered as a second-line vasopressor in patients with septic shock (1) and is no longer recommended as a vasopressor therapy in patients with hemorrhagic shock (86).

In patients with cardiogenic shock, there is no evidence of superiority of one vasopressor over another in terms of mortality (106). However, although epinephrine and the association norepinephrine and dobutamine has similar hemodynamic efficiency, patients with cardiogenic shock receiving epinephrine experience more lactic acidosis and arrhythmias and have inadequate gastric mucosa perfusion (107). More recently, Levy et al. (108) showed in a randomized trial including 57 patients with cardiogenic shock after myocardial infarction confirmed that epinephrine and norepinephrine had similar hemodynamic efficacy, but epinephrine was associated with higher incidence of refractory shock. Currently, it is recommended to favor norepinephrine over epinephrine in patients with cardiogenic shock requiring vasopressors (89).

#### Phenylephrine

Phenylephrine is a pure α-adrenergic receptor agonist with marked vasopressor activity (81, 93). The effects of phenylephrine on cardiac output are complex and difficult to predict, depending on its venous and arterial modulation (109). Phenylephrine-induced venous vasoconstriction might exert opposite effects on systemic venous return. On one hand, it decreases the unstressed venous volume, which in turn increases the mean systemic filling pressure, the venous return pressure gradient and thus the systemic venous return. On the other hand, it also increases the resistance to venous return, which consequently decreases the systemic venous return. Thus, phenylephrine may either increase or decrease cardiac output in patients with preload reserve (110). Moreover, phenylephrine-induced arterial vasoconstriction results in a marked increase in left ventricular afterload with an important rise in systolic arterial pressure and thus may induce a decrease in cardiac output in patients with impaired cardiac contractility (111). This marked increase in systolic arterial pressure may also result in baroreceptor-mediated reflex bradycardia and contribute to the decrease in cardiac output (81, 93). Recently, Kalmar et al. (112) showed in patients with preload reserve and anesthesia-induced hypotension that a single phenylephrine administration increased the systemic venous return and thus cardiac preload, which in turn increased cardiac output while the left ventricular afterload increased. Besides its hemodynamic effects, phenylephrine might also have some immune effects (85).

While phenylephrine is widely used to restore arterial pressure in anesthesia-induced hypotensive patients in the operating theater (113), its use is no longer recommended in critically-ill patients with septic shock from 2016 (1, 101). First, phenylephrine induced a more pronounced global α1-mediated splanchnic vasoconstriction than norepinephrine (114), with a potential risk of splanchnic ischemia (115), even though the adverse effects of phenylephrine on microcirculatory blood flow in the gastrointestinal tracts might be less marked than those of epinephrine and norepinephrine (20). Second, phenylephrine has a lower efficacy than norepinephrine when continuously administered due to the absence of β-adrenergic effects (116). Third, phenylephrine use could be associated with a higher mortality rate in critically-ill patients (117).

#### Dopamine

Dopamine is the immediate physiologic precursor of norepinephrine and epinephrine. Its effects depend on the activated receptors, which in turn depend on the dose administered (81, 118, 119). At low dose (<5 μg/kg/min), dopamine activates D1 receptors located in cerebral, coronary renal and mesenteric vessels and induces vasodilation with no effect on arterial pressures. At intermediate dose (5–10 μg/kg/min), dopamine has chronotropic and inotropic effects by activating the β1-adrenergic receptor. At high dose (10–20 μg/kg/min), by activating the α-adrenergic receptor, dopamine has a vasopressor activity similar to that of norepinephrine, with arterial and venous vasoconstriction, which results in an increase in systemic venous return and left ventricular afterload. As with other catecholaminergic vasopressors, dopamine might also have some immune effects (120, 121).

Regarding microcirculation, dopamine has similar effects than norepinephrine in splanchnic circulation (102). Yet, gastroduodenal motility was found to be adversely impacted by the use of low-dose dopamine (4 μg/kg per minute) as compared to placebo in mechanically ventilated critically ill patients (122). Currently, dopamine is no longer recommended in critically-ill patients for the following reasons. First, there is a great inter-individual variability of the dopamine effects, because of unpredictable relationship between infusion rate and plasma levels (81). Second, in a multicenter and randomized trial, De Backer et al. (123) compared dopamine to norepinephrine as first-line vasopressor therapy to restore and maintain blood pressure in 1,679 patients with shock. While there was no difference in mortality rate, except in the subgroup of patients with cardiogenic shock, dopamine was associated with a two-fold incidence of cardiac arrhythmias (123), confirming findings of a previous study conducted in patients with septic shock (124). Conversely, the incidence rate of ischemic complications (skin ischemia and arterial occlusions) with dopamine was similar to that observed with norepinephrine. In particular; AMI was reported in <1% of patients (123).

## Limitations

We acknowledge several and important biases due to the nature of the pharmacovigilance database. The first being underreporting, associated with halo bias and lack of information on the exposed population for calculation of incidence, which would require sales data from the industry. Indeed, acute mesenteric ischemia may be caused by shock, which is a major confusion bias. Moreover, as mesenteric ischemia remains a rare entity, true incidence remains elusive, due to numerous factors. The lack of consensual definition, with multiple criteria possible, as well as hardship to have definite diagnosis makes underreporting plausible. Moreover, not being able to return to each report to ensure that an exhaustive search for etiologies and concomitant drugs intake has been carried out leads to an information bias, which leads to the fact that the likelihood of a causal relationship is not the same in all reports. It is of importance to underline that the association we found between vasopressors and acute mesenteric ischemia is not necessarily synonymous with causality and, given the low quality of evidence that can be inferred from the analysis of large databases such as the pharmacovigilance database, our results should be interpret with caution. Yet, with all these elements, disproportionality analysis methodology allows to focus the attention of clinical physicians, and to assess plausibility of the incrimination of a drug toward a singular adverse effect. Although all vasopressors except Dopamine do not have dose-dependent physiological effects and were used in the same indications in the different studies included in our literature review, it should be kept in mind that the incidence of acute mesenteric ischemia may be influenced by the dose of vasopressors administered, especially in patients with impaired vascularity (elderly patients, smokers…). However, as the dose ranges of vasopressors used in the different studies are very similar, it is not possible to analyze a potential dose-dependent effect. The numerous models and mechanisms, yielded from *in vivo* models, which we presented in this review of literature bring support to these findings, and warrant further scrutiny in the field.

## Conclusion

In this pharmacovigilance analysis combined with literature review, we observed a significant association between the use of all vasopressors but not selepressin and AMI in patients with vasodilatory shock. The development of new-generation of vasopressors activating different receptors and intracellular pathways, the individualization of a vasopressor therapy based on specific biomarkers and the development of artificial intelligence to better adjust in real-time vasopressor therapy may help in the future to avoid vasopressor-related AMI in critically ill patients ad help improve the management of patients with vasodilatory shock.

## Data Availability Statement

Publicly available datasets were analyzed in this study. This data can be found here: www.vigiaccess.org.

## Author Contributions

CD allowed statistical analyses on VigiBase^®^ extraction. LN wrote the final manuscript and supervised the study. All authors actively participated to the manuscript writing and provided critical insight to its revision. All authors contributed to the article and approved the submitted version.

## Author Disclaimer

The information does not represent the opinion of World Health Organization.

## Conflict of Interest

The authors declare that the research was conducted in the absence of any commercial or financial relationships that could be construed as a potential conflict of interest.

## Publisher's Note

All claims expressed in this article are solely those of the authors and do not necessarily represent those of their affiliated organizations, or those of the publisher, the editors and the reviewers. Any product that may be evaluated in this article, or claim that may be made by its manufacturer, is not guaranteed or endorsed by the publisher.

## References

[1] Evans L Rhodes A Alhazzani W Antonelli M Coopersmith CM French C. Surviving sepsis campaign: international guidelines for management of sepsis and septic shock 2021. Intensive Care Med. (2021) 47:1181–247. 10.1007/s00134-021-06506-y 34599691 PMC8486643

[2] Leone M Bechis C Baumstarck K Ouattara A Collange O Augustin P. Outcome of acute mesenteric ischemia in the intensive care unit: a retrospective, multicenter study of 780 cases. Intensive Care Med. (2015) 41:667–76. 10.1007/s00134-015-3690-8 25731634

[3] Duran M Pohl E Grabitz K Schelzig H Sagban TA Simon F. The importance of open emergency surgery in the treatment of acute mesenteric ischemia. World J Emerg Surg. (2015) 10:45. 10.1186/s13017-015-0041-6 26413147 PMC4583757

[4] Bourcier S Klug J Nguyen LS. Non-occlusive mesenteric ischemia: Diagnostic challenges and perspectives in the era of artificial intelligence. World J Gastroenterol. (2021) 27:4088–103. 10.3748/wjg.v27.i26.4088 34326613 PMC8311528

[5] Lindquist M. Data quality management in pharmacovigilance. Drug safety. (2004) 27:857–70. 10.2165/00002018-200427120-00003 15366974

[6] M.M.a.S.S. Organization. Introductory Guide to MedDRA. M.M.a.S.S. Organization (2022).

[7] Johnson DB Balko JM Compton ML Chalkias S Gorham J Xu Y. Fulminant myocarditis with combination immune checkpoint blockade. N Engl J Med. (2016) 375:1749–55. 10.1056/NEJMoa1609214 27806233 PMC5247797

[8] Salem JE Manouchehri A Moey M Lebrun-Vignes B Bastarache L Pariente A. Cardiovascular toxicities associated with immune checkpoint inhibitors: an observational, retrospective, pharmacovigilance study. Lancet Oncol. (2018) 19:1579–89. 10.1016/S1470-2045(18)30608-9 30442497 PMC6287923

[9] Bate A Lindquist M Edwards IR Olsson S Orre R Lansner A. A Bayesian neural network method for adverse drug reaction signal generation. Eur J Clin Pharmacol. (1998) 54:315–21. 10.1007/s002280050466 9696956

[10] Norén GN Hopstadius J Bate A. Shrinkage observed-to-expected ratios for robust and transparent large-scale pattern discovery. Stat Methods Med Res. (2013) 22:57–69. 10.1177/0962280211403604 21705438 PMC6331976

[11] Salem JE Allenbach Y Vozy A Brechot N Johnson DB Moslehi JJ. Abatacept for severe immune checkpoint inhibitor-associated myocarditis. N Engl J Med. (2019) 380:2377–9. 10.1056/NEJMc1901677 31189043

[12] Salem JE Manouchehri A Bretagne M Lebrun-Vignes B Groarke JD Johnson DB. Cardiovascular toxicities associated with ibrutinib. J Am Coll Cardiol. (2019) 74:1667–78. 10.1016/j.jacc.2019.07.056 31558250

[13] Salem JE Yang T Moslehi JJ Waintraub X Gandjbakhch E Bachelot A. Androgenic effects on ventricular repolarization: a translational study from the international pharmacovigilance database to iPSC-cardiomyocytes. Circulation. (2019) 140:1070–80. 10.1161/CIRCULATIONAHA.119.040162 31378084 PMC6756939

[14] Salem JE Nguyen LS Moslehi JJ Ederhy S Lebrun-Vignes B Roden DM. Anticancer drug-induced life-threatening ventricular arrhythmias: a World Health Organization pharmacovigilance study. Eur Heart J. (2021). 10.1093/eurheartj/ehab362 34370839 PMC8677441

[15] Nguyen LS Dolladille C Drici MD Fenioux C Alexandre J Mira JP. Cardiovascular toxicities associated with hydroxychloroquine and azithromycin: an analysis of the world health organization pharmacovigilance database. Circulation. (2020) 142:303–5. 10.1161/CIRCULATIONAHA.120.048238 32442023 PMC7365677

[16] Wisniewski AF Bate A Bousquet C Brueckner A Candore G Juhlin K. Good signal detection practices: evidence from IMI PROTECT. Drug safety. (2016) 39:469–90. 10.1007/s40264-016-0405-1 26951233 PMC4871909

[17] Dargent A Nguyen M Fournel I Bourredjem A Charles PE Quenot JP. Vasopressor cumulative dose requirement and risk of early death during septic shock: an analysis from the EPISS cohort. Shock. (2018) 49:625–30. 10.1097/SHK.0000000000001022 29040212

[18] Prys-Picard CO Shah SK Williams BD Cardenas V Jr Sharma G. Outcomes of patients on multiple vasoactive drugs for shock. J Intensive Care Med. (2013) 28:237–40. 10.1177/0885066612448738 22733722

[19] El Moheb M Naar L Christensen MA Kapoen C Maurer LR Farhat M. Gastrointestinal complications in critically ill patients with and without COVID-19. Jama. (2020) 324:1899–901. 10.1001/jama.2020.19400 32970139 PMC7516799

[20] Krejci V Hiltebrand LB Sigurdsson GH. Effects of epinephrine, norepinephrine, and phenylephrine on microcirculatory blood flow in the gastrointestinal tract in sepsis. Crit Care Med. (2006) 34:1456–63. 10.1097/01.CCM.0000215834.48023.57 16557162

[21] Silva E DeBacker D Créteur J Vincent JL. Effects of vasoactive drugs on gastric intramucosal pH. Crit Care Med. (1998) 26:1749–58. 10.1097/00003246-199810000-00034 9781735

[22] van der Spoel JI Schultz MJ van der Voort PH de Jonge E. Influence of severity of illness, medication and selective decontamination on defecation. Intensive Care Med. (2006) 32:875–80. 10.1007/s00134-006-0175-9 16715327

[23] Guillaume A Pili-Floury S Chocron S Delabrousse E De Parseval B Koch S. Acute mesenteric ischemia among postcardiac surgery patients presenting with multiple organ failure. Shock (2017) 47:296–302. 10.1097/SHK.0000000000000720 28195969

[24] Brennan CA Osei-Bonsu P McClenaghan RE Nassar A Forget P Kaye C. Vasoactive agents in acute mesenteric ischaemia in critical care. A systematic review. F1000Res. (2021) 10:453. 10.12688/f1000research.52782.1 34621507 PMC8459625

[25] De Gasparo M Catt KJ Inagami T Wright JW Unger T. International union of pharmacology. XXIII. The angiotensin II receptors. Pharmacol Rev. (2000) 52:415–72. 10977869

[26] Laghlam D Jozwiak M Nguyen LS. Renin-angiotensin-aldosterone system and immunomodulation: a state-of-the-art review. Cells. (2021) 10:1767. 10.3390/cells10071767 34359936 PMC8303450

[27] Basso N Terragno NA. History about the discovery of the renin-angiotensin system. Hypertension. (2001) 38:1246–9. 10.1161/hy1201.101214 11751697

[28] Wan L Langenberg C Bellomo R May CN. Angiotensin II in experimental hyperdynamic sepsis. Crit Care. (2009) 13:R190. 10.1186/cc8185 19948019 PMC2811902

[29] May CN Ishikawa K Wan L Williams J Wellard RM Pell GS. Renal bioenergetics during early gram-negative mammalian sepsis and angiotensin II infusion. Intensive Care Med. (2012) 38:886–93. 10.1007/s00134-012-2487-2 22302028

[30] Corrêa TD Jeger V Pereira AJ Takala J Djafarzadeh S Jakob SM. Angiotensin II in septic shock: effects on tissue perfusion, organ function, and mitochondrial respiration in a porcine model of fecal peritonitis. Crit Care Med. (2014) 42:e550–9. 10.1097/CCM.0000000000000397 24797374

[31] Wray GM Coakley JH. Severe septic shock unresponsive to noradrenaline. Lancet. (1995) 346:1604. 10.1016/S0140-6736(95)91933-3 7500755

[32] Del Greco F Johnson DC. Clinical experience with angiotensin II in the treatment of shock. JAMA. (1961) 178:994–9. 10.1001/jama.1961.03040490020005 13884972

[33] Derrick JR Anderson JR Roland BJ. Adjunctive use of a biologic pressor agent, angiotensin, in management of shock. Circulation. (1962) 25:263–7. 10.1161/01.CIR.25.1.263 13885617

[34] Antonucci E Gleeson PJ Annoni F Agosta S Orlando S Taccone FS. Angiotensin II in refractory septic shock. Shock. (2017) 47:560–6. 10.1097/SHK.0000000000000807 27879559

[35] Thomas VL Nielsen MS. Administration of angiotensin II in refractory septic shock. Crit Care Med. (1991) 19:1084–6. 10.1097/00003246-199108000-00020 1860334

[36] Chawla LS Busse L Brasha-Mitchell E Davison D Honiq J Alotaibi Z. Intravenous angiotensin II for the treatment of high-output shock (ATHOS trial): a pilot study. Crit Care. (2014) 18:534. 10.1186/s13054-014-0534-9 25286986 PMC4212099

[37] Khanna A English SW Wang XS Ham K Tumlin J Szerlip H. Angiotensin II for the treatment of vasodilatory shock. N Engl J Med. (2017) 377:419–30. 10.1056/NEJMoa1704154 28528561

[38] Wakefield BJ Sacha GL Khanna AK. Vasodilatory shock in the ICU and the role of angiotensin II. Curr Opin Crit Care. (2018) 24:277–85. 10.1097/MCC.0000000000000517 29877879

[39] Bellomo R Forni LG Busse LW McCurdy MT Ham KR Boldt DW. Renin and survival in patients given angiotensin ii for catecholamine-resistant vasodilatory shock: a clinical trial. Am J Respir Crit Care Med. (2020) 202:1253–61. 10.1164/rccm.201911-2172OC 32609011 PMC7605187

[40] Tumlin JA Murugan R Deane AM Ostermann M Busse LW Ham KR. Outcomes in patients with vasodilatory shock and renal replacement therapy treated with intravenous angiotensin II. Crit Care Med. (2018) 46:949–57. 10.1097/CCM.0000000000003092 29509568 PMC5959265

[41] Laesser M Oi Y Ewert S Fändriks L Aneman A. The angiotensin II receptor blocker candesartan improves survival and mesenteric perfusion in an acute porcine endotoxin model. Acta Anaesthesiol Scand. (2004) 48:198–204. 10.1111/j.0001-5172.2004.00283.x 14995942

[42] Nitescu N Grimberg E Guron G. Low-dose candesartan improves renal blood flow and kidney oxygen tension in rats with endotoxin-induced acute kidney dysfunction. Shock. (2008) 30:166–72. 10.1097/SHK.0b013e31815dd780 18091574

[43] Busse LW Wang XS Chalikonda DM Finkel KW Khanna AK Szerlip HM. Clinical experience with IV angiotensin II administration: a systematic review of safety. Crit Care Med. (2017) 45:1285–94. 10.1097/CCM.0000000000002441 28489648 PMC5515638

[44] Demiselle J Fage N Radermacher P Asfar P. Vasopressin and its analogues in shock states: a review. Ann Intensive Care. (2020) 10:9. 10.1186/s13613-020-0628-2 31970567 PMC6975768

[45] Barrett LK Singer M Clapp LH. Vasopressin: mechanisms of action on the vasculature in health and in septic shock. Crit Care Med. (2007) 35:33–40. 10.1097/01.CCM.0000251127.45385.CD 17133186

[46] Laporte R Kohan A Heitzmann J Wisniewska H Toy J La E. Pharmacological characterization of FE 202158, a novel, potent, selective, and short-acting peptidic vasopressin V1a receptor full agonist for the treatment of vasodilatory hypotension. J Pharmacol Exp Ther. (2011) 337:786–96. 10.1124/jpet.111.178848 21411496

[47] Möhring J Glänzer K Maciel JA Jr Düsing R Kramer HJ Arbogast R. Greatly enhanced pressor response to antidiuretic hormone in patients with impaired cardiovascular reflexes due to idiopathic orthostatic hypotension. J Cardiovasc Pharmacol. (1980) 2:367–76. 10.1097/00005344-198007000-00004 6156335

[48] Graybiel A Glendy RE. Circulatory effects following the intravenous administration of pitressin in normal persons and in patients with hypertension and angina pectoris. Am Heart J. (1941) 21:481–9. 10.1016/S0002-8703(41)90649-X

[49] Wilson MF Brackett DJ Hinshaw LB Tompkins P Archer LT Benjamin BA. Vasopressin release during sepsis and septic shock in baboons and dogs. Surg Gynecol Obstet. (1981) 153:869–72. 7029759

[50] Brackett DJ Schaefer CF Tompkins P Fagraeus L Peters LJ Wilson MF. Evaluation of cardiac output, total peripheral vascular resistance, and plasma concentrations of vasopressin in the conscious, unrestrained rat during endotoxemia. Circ Shock. (1985) 17:273–84. 4092342

[51] Landry DW Levin HR Gallant EM Ashton RC Jr Seo S D'Alessandro D. Vasopressin deficiency contributes to the vasodilation of septic shock. Circulation. (1997) 95:1122–5. 10.1161/01.CIR.95.5.1122 9054839

[52] Sharshar T Carlier R Blanchard A Feydy A Gray F Paillard M. Depletion of neurohypophyseal content of vasopressin in septic shock. Crit Care Med. (2002) 30:497–500. 10.1097/00003246-200203000-00001 11990905

[53] Sharshar T Blanchard A Paillard M Raphael JC Gajdos P Annane D. Circulating vasopressin levels in septic shock. Crit Care Med. (2003) 31:1752–8. 10.1097/01.CCM.0000063046.82359.4A 12794416

[54] Annane D Trabold F Sharshar T Jarrin I Blanc AS Raphael JC. Inappropriate sympathetic activation at onset of septic shock: a spectral analysis approach. Am J Respir Crit Care Med. (1999) 160:458–65. 10.1164/ajrccm.160.2.9810073 10430714

[55] Garrard CS Kontoyannis DA Piepoli M. Spectral analysis of heart rate variability in the sepsis syndrome. Clin Auton Res. (1993) 3:5–13. 10.1007/BF01819137 8386574

[56] Siami S Bailly-Salin J Polito A Porcher R Blanchard A Haymann JP. Osmoregulation of vasopressin secretion is altered in the postacute phase of septic shock. Crit Care Med. (2010) 38:1962–9. 10.1097/CCM.0b013e3181eb9acf 20639747

[57] Sharshar T Gray F Lorin G de la Grandmaison NS Hopkinson E Ross A. Apoptosis of neurons in cardiovascular autonomic centres triggered by inducible nitric oxide synthase after death from septic shock. Lancet. (2003) 362:1799–805. 10.1016/S0140-6736(03)14899-4 14654318

[58] Sonneville R Guidoux C Barrett L Viltart O Mattot V Polito A. Vasopressin synthesis by the magnocellular neurons is different in the supraoptic nucleus and in the paraventricular nucleus in human and experimental septic shock. Brain Pathol. (2010) 20:613–22. 10.1111/j.1750-3639.2009.00355.x 20015289 PMC8094829

[59] Liard JF Dériaz O Schelling P Thibonnier M. Cardiac output distribution during vasopressin infusion or dehydration in conscious dogs. Am J Physiol. (1982) 243:H663–9. 10.1152/ajpheart.1982.243.5.H663 7137358

[60] Bomberg H Bierbach B Flache S Novák M Schäfers HJ Menger MD. Dobutamine versus vasopressin after mesenteric ischemia. J Surg Res. (2019) 235:410–23. 10.1016/j.jss.2018.10.028 30691823

[61] Vercauteren M Strasser D Vezzali E Stalder A Iglarz M Hess P. Vasopressin is involved in endothelin receptor antagonist-induced fluid retention in rat. Differential effect of selective ETA and dual ETA/ETB receptor antagonists. Eur Resp J. (2012) 40:3898. 10.1124/jpet.116.234930 28223322

[62] Russell JA Walley KR Singer J Gordon AC Hébert PC Cooper DJ. Vasopressin versus norepinephrine infusion in patients with septic shock. N Engl J Med. (2008) 358:877–87. 10.1056/NEJMoa067373 18305265

[63] Jiang L Sheng Y Feng X Wu J. The effects and safety of vasopressin receptor agonists in patients with septic shock: a meta-analysis and trial sequential analysis. Crit Care. (2019) 23:91. 10.1186/s13054-019-2362-4 30871607 PMC6419432

[64] Wang H Liu A Bo W Feng X Hu Y. Terlipressin in the treatment of hepatorenal syndrome: a systematic review and meta-analysis. Medicine (Baltimore). (2018) 97:e0431. 10.1097/MD.0000000000010431 29668606 PMC5916651

[65] Döhler KD Meyer M. Vasopressin analogues in the treatment of hepatorenal syndrome and gastrointestinal haemorrhage. Best Pract Res Clin Anaesthesiol. (2008) 22:335–50. 10.1016/j.bpa.2008.02.002 18683479

[66] Liu ZM Chen J Kou Q Lin Q Huang X Tang Z. Terlipressin versus norepinephrine as infusion in patients with septic shock: a multicentre, randomised, double-blinded trial. Intensive Care Med. (2018) 44:1816–25. 10.1007/s00134-018-5267-9 29971593

[67] Huang P Guo Y Li B Liu Q. Terlipressin versus norepinephrine for septic shock: a systematic review and meta-analysis. Front Pharmacol. (2019) 10:1492. 10.3389/fphar.2019.01492 31920672 PMC6936170

[68] Yao RQ Xia DM Wang LX Wu GS Zhu YB Zhao HQ. Clinical efficiency of vasopressin or its analogs in comparison with catecholamines alone on patients with septic shock: a systematic review and meta-analysis. Front Pharmacol. (2020) 11:563. 10.3389/fphar.2020.00563 32435192 PMC7218087

[69] He X Su F Taccone FS Laporte R Kjølbye AL Zhang J. A selective V(1A) receptor agonist, selepressin, is superior to arginine vasopressin and to norepinephrine in ovine septic shock. Crit Care Med. (2016) 44:23–31. 10.1097/CCM.0000000000001380 26496451 PMC4684247

[70] Maybauer MO Maybauer DM Enkhbaatar P Laporte R Wiśniewska H Traber LD. The selective vasopressin type 1a receptor agonist selepressin (FE 202158) blocks vascular leak in ovine severe sepsis^*^. Crit Care Med. (2014) 42:e525–33. 10.1097/CCM.0000000000000300 24674922 PMC4346299

[71] Rehberg S Yamamoto Y Sousse L Bartha E Jonkam C Hasselbach AK. Selective V(1a) agonism attenuates vascular dysfunction and fluid accumulation in ovine severe sepsis. Am J Physiol Heart Circ Physiol. (2012) 303:H1245–54. 10.1152/ajpheart.00390.2012 22961865 PMC3517638

[72] Rehberg S Ertmer C Vincent JL Morelli A Schneider M Lange M. Role of selective V1a receptor agonism in ovine septic shock. Crit Care Med. (2011) 39:119–25. 10.1097/CCM.0b013e3181fa3898 20890184

[73] Barabutis N Marinova M Solopov P Uddin MA Croston GE Reinheimer TM. Protective mechanism of the selective vasopressin V(1A) receptor agonist selepressin against endothelial barrier dysfunction. J Pharmacol Exp Ther. (2020) 375:286–95. 10.1124/jpet.120.000146 32943478

[74] Russell JA Vincent JL Kjølbye AL Olsson H Blemings A Spapen H. Selepressin, a novel selective vasopressin V(1A) agonist, is an effective substitute for norepinephrine in a phase IIa randomized, placebo-controlled trial in septic shock patients. Crit Care. (2017) 21:213. 10.1186/s13054-017-1798-7 28807037 PMC5557574

[75] Laterre PF Berry SM Blemings A Carlsen JE François B Graves T. Effect of selepressin vs placebo on ventilator- and vasopressor-free days in patients with septic shock: the SEPSIS-ACT randomized clinical trial. JAMA. (2019) 322:1476–85. 10.1001/jama.2019.14607 31577035 PMC6802260

[76] Hamzaoui O Georger JF Monnet X Ksouri H Maizel J Richard C. Early administration of norepinephrine increases cardiac preload and cardiac output in septic patients with life-threatening hypotension. Crit Care. (2010) 14:R142. 10.1186/cc9207 20670424 PMC2945123

[77] Monnet X Jabot J Maizel J Richard C Teboul JL. Norepinephrine increases cardiac preload and reduces preload dependency assessed by passive leg raising in septic shock patients. Crit Care Med. (2011) 39:689–94. 10.1097/CCM.0b013e318206d2a3 21263328

[78] Persichini R Silva S Teboul JL Jozwiak M Chemla D Richard C. Effects of norepinephrine on mean systemic pressure and venous return in human septic shock. Crit Care Med. (2012) 40:3146–53. 10.1097/CCM.0b013e318260c6c3 22926333

[79] Adda I Lai C Teboul JL Guerin L Gavelli F Monnet X. Norepinephrine potentiates the efficacy of volume expansion on mean systemic pressure in septic shock. Crit Care. (2021) 25:302. 10.1186/s13054-021-03711-5 34419120 PMC8379760

[80] Hamzaoui O Jozwiak M Geffriaud T Sztrymf B Prat D Jacobs F. Norepinephrine exerts an inotropic effect during the early phase of human septic shock. Br J Anaesth. (2018) 120:517–24. 10.1016/j.bja.2017.11.065 29452808

[81] Russell JA Gordon AC Williams MD Boyd JH Walley KR Kissoon N. Vasopressor therapy in the intensive care unit. Semin Respir Crit Care Med. (2021) 42:59–77. 10.1055/s-0040-1710320 32820475

[82] Backer D Creteur J Preiser JC Dubois MJ Vincent JL. Microvascular blood flow is altered in patients with sepsis. Am J Respir Crit Care Med. (2002) 166:98–104. 10.1164/rccm.200109-016OC 12091178

[83] Georger JF Hamzaoui O Chaari A Maizel J Richard C Teboul JL. Restoring arterial pressure with norepinephrine improves muscle tissue oxygenation assessed by near-infrared spectroscopy in severely hypotensive septic patients. Intensive Care Med. (2010) 36:1882–9. 10.1007/s00134-010-2013-3 20689910

[84] Jozwiak M Chambaz M Sentenac P Monnet X Teboul JL. Assessment of tissue oxygenation to personalize mean arterial pressure target in patients with septic shock. Microvasc Res. (2020) 132:104068. 10.1016/j.mvr.2020.104068 32877698

[85] Stolk RF van der Poll T Angus DC van der Hoeven JG Pickkers P Kox M. Potentially inadvertent immunomodulation: norepinephrine use in sepsis. Am J Respir Crit Care Med. (2016) 194:550–8. 10.1164/rccm.201604-0862CP 27398737

[86] Spahn DR Bouillon B Cerny V Duranteau J Filipescu D Hunt BJ. The European guideline on management of major bleeding and coagulopathy following trauma: fifth edition. Crit Care. (2019) 23:98. 10.1186/s13054-019-2347-3 30917843 PMC6436241

[87] Levy B Bastien O Karim B Cariou A Chouihed T Combes A. Experts' recommendations for the management of adult patients with cardiogenic shock. Ann Intensive Care. (2015) 5:52. 10.1186/s13613-015-0052-1 26152849 PMC4495097

[88] Møller MH Claudius C Junttila E Haney M Oscarsson-Tibblin A Haavind A. Scandinavian SSAI clinical practice guideline on choice of first-line vasopressor for patients with acute circulatory failure. Acta Anaesthesiol Scand. (2016) 60:1347–66. 10.1111/aas.12780 27576362 PMC5213738

[89] McDonagh TA Metra M Adamo M Gardner RS Baumbach A Böhm M. ESC Guidelines for the diagnosis and treatment of acute and chronic heart failure. Eur Heart J. (2012) 42:3599–726. 10.1093/eurheartj/ehab368 34447992

[90] Teboul JL Duranteau J Russell JA. Intensive care medicine in 2050: vasopressors in sepsis. Intensive Care Med. (2018) 44:1130–2. 10.1007/s00134-017-4909-7 28861671

[91] Seilitz J Grafver I Kiszakiewicz L Oikonomakis I Jansson K Axelsson B. A randomized porcine study in low cardiac output of vasoactive and inotropic drug effects on the gastrointestinal tract. Shock. (2021) 56:308–17. 10.1097/SHK.0000000000001726 33443363 PMC8529897

[92] Asfar P Meziani F Hamel JF Grelon F Megarbane B Anguel N. High versus low blood-pressure target in patients with septic shock. N Engl J Med. (2014) 370:1583–93. 10.1056/NEJMoa1312173 24635770

[93] Russell JA. Vasopressor therapy in critically ill patients with shock. Intensive Care Med. (2019) 45:1503–17. 10.1007/s00134-019-05801-z 31646370

[94] Myburgh JA Higgins A Jovanovska A Lipman J Ramakrishnan N Santamaria J. A comparison of epinephrine and norepinephrine in critically ill patients. Intensive Care Med. (2008) 34:2226–34. 10.1007/s00134-008-1219-0 18654759

[95] Annane D Vignon P Renault A Bollaert PE Charpentier C Martin C. Norepinephrine plus dobutamine versus epinephrine alone for management of septic shock: a randomised trial. Lancet. (2007) 370:676–84. 10.1016/S0140-6736(07)61344-0 17720019

[96] Levy B Bollaert PE Charpentier C Nace L Audibert G Bauer P. Comparison of norepinephrine and dobutamine to epinephrine for hemodynamics, lactate metabolism, and gastric tonometric variables in septic shock: a prospective, randomized study. Intensive Care Med. (1997) 23:282–7. 10.1007/s001340050329 9083230

[97] Levy B Mansart A Bollaert PE Franck P Mallie JP. Effects of epinephrine and norepinephrine on hemodynamics, oxidative metabolism, and organ energetics in endotoxemic rats. Intensive Care Med. (2003) 29:292–300. 10.1007/s00134-002-1611-0 12594589

[98] Levy B Desebbe O Montemont C Gibot S. Increased aerobic glycolysis through beta2 stimulation is a common mechanism involved in lactate formation during shock states. Shock. (2008) 30:417–21. 10.1097/SHK.0b013e318167378f 18323749

[99] Levy B Gibot S Franck P Cravoisy A Bollaert PE. Relation between muscle Na+K+ ATPase activity and raised lactate concentrations in septic shock: a prospective study. Lancet. (2005) 365:871–5. 10.1016/S0140-6736(05)71045-X 15752531

[100] Duranteau J Sitbon P Teboul JL Vicaut E Anguel N Richard C. Effects of epinephrine, norepinephrine, or the combination of norepinephrine and dobutamine on gastric mucosa in septic shock. Crit Care Med. (1999) 27:893–900. 10.1097/00003246-199905000-00021 10362410

[101] Rhodes A Evans LE Alhazzani W Levy MM Antonelli M Ferrer R. Surviving sepsis campaign: international guidelines for management of sepsis and septic shock: 2016. Crit Care Med. (2017) 45:486–552. 10.1097/CCM.0000000000002255 28098591

[102] de Backer D Creteur J Silva E Vincent JL. Effects of dopamine, norepinephrine, and epinephrine on the splanchnic circulation in septic shock: which is best? Crit Care Med. (2003) 31:1659–67. 10.1097/01.CCM.0000063045.77339.B6 12794401

[103] Levy B Bollaert PE Lucchelli JP Sadoune LO Nace L Larcan A. Dobutamine improves the adequacy of gastric mucosal perfusion in epinephrine-treated septic shock. Crit Care Med. (1997) 25:1649–54. 10.1097/00003246-199710000-00013 9377878

[104] Zhou SX Qiu HB Huang YZ Yang Y Zheng RQ. Effects of norepinephrine, epinephrine, and norepinephrine-dobutamine on systemic and gastric mucosal oxygenation in septic shock. Acta Pharmacol Sin. (2002) 23:654–8. 12100762

[105] Fruhwald S Scheidl S Toller W Petnehazy T Holzer P Metzler H. Low potential of dobutamine and dopexamine to block intestinal peristalsis as compared with other catecholamines. Crit Care Med. (2000) 28:2893–7. 10.1097/00003246-200008000-00034 10966267

[106] Levy B Klein T Kimmoun A. Vasopressor use in cardiogenic shock. Curr Opin Crit Care. (2020) 26:411–6. 10.1097/MCC.0000000000000743 32487842

[107] Levy B Perez P Perny J Thivilier C Gerard A. Comparison of norepinephrine-dobutamine to epinephrine for hemodynamics, lactate metabolism, and organ function variables in cardiogenic shock. A prospective, randomized pilot study. Crit Care Med. (2011) 39:450–5. 10.1097/CCM.0b013e3181ffe0eb 21037469

[108] Levy B Clere-Jehl R Legras A Morichau-Beauchant T Leone M Frederique G. Epinephrine versus norepinephrine for cardiogenic shock after acute myocardial infarction. J Am Coll Cardiol. (2018) 72:173–82. 10.1016/j.jacc.2018.04.051 29976291

[109] Jozwiak M Rex S Bendjelid K. Boosting systemic pressure with phenylephrine: arterial or venous modulation? J Clin Monit Comput. (2018) 32:967–8. 10.1007/s10877-018-0177-5 29959580

[110] Rebet O Andremont O Gérard JL Fellahi JL Hanouz JL Fischer MO. Preload dependency determines the effects of phenylephrine on cardiac output in anaesthetised patients: a prospective observational study. Eur J Anaesthesiol. (2016) 33:638–44. 10.1097/EJA.0000000000000470 27164015

[111] Robotham JL Takata M Berman M Harasawa Y. Ejection fraction revisited. Anesthesiology. (1991) 74:172–83. 10.1097/00000542-199101000-00026 1986643

[112] Kalmar AF Allaert S Pletinckx P Maes JW Heerman J Vos JJ. Phenylephrine increases cardiac output by raising cardiac preload in patients with anesthesia induced hypotension. J Clin Monit Comput. (2018) 32:969–76. 10.1007/s10877-018-0126-3 29569112 PMC6209056

[113] Goertz AW Schmidt M Seefelder C Lindner KH Georgieff M. The effect of phenylephrine bolus administration on left ventricular function during isoflurane-induced hypotension. Anesth Analg. (1993) 77:227–31. 10.1213/00000539-199308000-00004 8346819

[114] Nygren A Thorén A Ricksten SE. Vasopressors and intestinal mucosal perfusion after cardiac surgery: norepinephrine vs. phenylephrine. Crit Care Med. (2006) 34:722–9. 10.1097/01.CCM.0000201879.20281.C6 16505658

[115] Zhou F Mao Z Zeng X Kang H Liu H Pan L. Vasopressors in septic shock: a systematic review and network meta-analysis. Ther Clin Risk Manag. (2015) 11:1047–59. 10.2147/TCRM.S80060 26203253 PMC4508075

[116] Morelli A Ertmer C Rehberg S Lange M Orecchioni A Laderchi A. Phenylephrine versus norepinephrine for initial hemodynamic support of patients with septic shock: a randomized, controlled trial. Crit Care. (2008) 12:R143. 10.1186/cc7121 19017409 PMC2646303

[117] Vail E Gershengorn HB Hua M Walkey AJ Rubenfeld G Wunsch H. Association between US norepinephrine shortage and mortality among patients with septic shock. JAMA. (2017) 317:1433–42. 10.1001/jama.2017.2841 28322415

[118] Allwood MJ Ginsburg J. Peripheral vascular and other effects of dopamine infusions in man. Clin Sci. (1964) 27:271–81. 14220906

[119] D'Orio VD Juchmès J Marcelle R. The use of low doses of *dopamine in intensive care medicine Arch Int Physiol Biochim*. (1984) 92:S11–20. 10.3109/13813458409071158 6085236

[120] Van den Berghe G de Zegher F. Anterior pituitary function during critical illness and dopamine treatment. Crit Care Med. (1996) 24:1580–90. 10.1097/00003246-199609000-00024 8797634

[121] Matt SM Gaskill PJ. Where is dopamine and how do immune cells see it?: Dopamine-mediated immune cell function in health and disease. J Neuroimmune Pharmacol. (2020) 15:114–64. 10.1007/s11481-019-09851-4 31077015 PMC6842680

[122] Dive A Foret F Jamart J Bulpa P Installé E. Effect of dopamine on gastrointestinal motility during critical illness. Intensive Care Med. (2000) 26:901–7. 10.1007/s001340051279 10990104

[123] Backer D Biston P Devriendt J Madl C Chochrad D Aldecoa C. Comparison of dopamine and norepinephrine in the treatment of shock. New Engl J Med. (2010). 362:779–89. 10.1056/NEJMoa0907118 20200382

[124] Patel GP Grahe JS Sperry M Singla S Elpern E Lateef O. Efficacy and safety of dopamine versus norepinephrine in the management of septic shock. Shock. (2010) 33:375–80. 10.1097/SHK.0b013e3181c6ba6f 19851126

